# DEX: a consensus-based amino acid exchangeability measure for improved codon substitution modelling

**DOI:** 10.64898/2026.03.09.710665

**Published:** 2026-03-12

**Authors:** Gavin M. Douglas, Louis-Marie Bobay

**Affiliations:** 1 Department of Biology, University of New Brunswick, Fredericton, New Brunswick, Canada; 2 Department of Biological Sciences, North Carolina State University, Raleigh, North Carolina, USA; 3 Bioinformatics Research Center, North Carolina State University, Raleigh, North Carolina, USA

## Abstract

Physicochemically similar amino acids undergo more frequent substitutions compared to dissimilar amino acid pairs. Despite their clear potential, amino acid similarity matrices remain underused in molecular evolution, partially due to the high number of proposed amino acid distance measures and the lack of agreement on which are most accurate. In this study, we assessed the performance of 30 amino acid distance measures, including a new amino acid distance measure we developed based on recent deep mutational scanning data. We compared these measures across codon substitution models fit to alignments spanning *Streptococcus*, *Drosophila*, and mammalian lineages, as well as segregating variants across *Escherichia coli* strains and human genotypes. We further constructed consensus measures from combinations of top-performing measures in this analysis using the DISTATIS approach and retested these matrices. Our results show that experimentally-derived measures, particularly our new measure and the existing experimental exchangeability (EX) measure, best fit codon substitution patterns across diverse lineages. We found that a consensus measure based on these two approaches, which we named DEX, performed best overall. In addition, although site-specific variant effect predictors are intended to identify deleterious mutations, the representative tools we tested did not outperform amino acid distance measures for predicting mean substitution frequencies. They were however substantially more informative for identifying individual highly deleterious mutations. Overall, we provide a systematic comparison of the performance of existing measures, and we introduce an improved general-purpose amino acid distance measure for molecular evolution models.

## Introduction

Amino acid substitutions have long been known to be more frequent between physicochemically similar amino acids relative to dissimilar pairs (Zuckerkandl and Pauling 1965; Dayhoff et al. 1978; Graur 1985). Nonetheless, amino acid distances are often ignored or underused when testing for selection based on non-synonymous substitution patterns. One key reason why amino acid distances remain underused in this context is that many contrasting measures have been proposed to summarize physicochemical properties and other characteristics. A comparison of amino acid distance measures is needed to assess which are most generalizable and useful for molecular evolution methods.

A standard test for natural selection acting on protein-coding DNA alignments is based on the ratio of non-synonymous to synonymous substitution rates (d_N_/d_S_) (Muse 1996). Early forms of this test explicitly incorporated amino acid distances (Miyata and Yasunaga 1980; Li et al. 1985; Goldman and Yang 1994), but the most common workflows now treat all amino acid substitutions as equally likely. A complementary approach is the ratio of radical to conservative amino substitutions (d_R_/d_C_) (Hughes et al. 1990; Zhang 2000). This approach is based on binning amino acid substitutions into those that occur between highly dissimilar amino acids (radical) and similar amino acids (conservative). Although d_R_/d_C_ is a valuable approach, particularly when corrected for compositional biases (Dagan et al. 2002; Weber and Whelan 2019), it relies on the simplistic binary categorization of substitutions despite continuous distances better capturing the relative impact of the different types of substitutions (James and Lascoux 2025). Both approaches, whether based on explicitly incorporating amino acid distances, or binning substitutions into groups, require accurate amino acid distance matrices to be effective. We hypothesize that methods incorporating continuous amino acid measures, which have been understudied in molecular evolution, could better estimate selection efficacy. However, understanding the relative utility of different amino acid distance measures is a necessary first step, and is our focus herein.

The most commonly used amino acid distance measures in molecular evolution are Grantham’s (Grantham 1974) and Miyata’s distances (Miyata et al. 1979). These measures are based on the volume and polarity of amino acids, as well as their chemical composition in the case of Grantham’s distance. Such measures are preferred over empirical distances, such as BLOSUM substitution matrices (Henikoff and Henikoff 1992), for modelling substitutions as the latter include mutational biases. Yet many other amino acid distance measures, and broader characteristics, that exclude mutational biases have been proposed, which warrant investigation for their relative utility in capturing non-synonymous substitution patterns.

Among the most promising of these measures are those based on mutational scanning data: experiments where many amino acid replacements are tested in the same protein to evaluate their phenotype independently from mutation biases. Early forms of these experiments were used to develop the experimental exchangeability (EX) measure (Yampolsky and Stoltzfus 2005). In recent years, several deep mutational scanning experiments have been conducted, where every possible individual amino acid replacement is tested at each site in a protein, and across a wider set of proteins (Fowler and Fields 2014; Notin et al. 2023). Importantly, these amino acid substitutions are performed systematically, and are not based on random mutagenesis, meaning that this approach does not incorporate mutational biases. These new datasets raise the possibility to update experimental exchangeability measures based on more extensive experimental data (Dunham and Beltrao 2021; Munro and Singh 2021).

Although amino acid distance measures warrant further investigation, these measures, by themselves, are ultimately simplistic. Indeed, some sites of a protein sequence are more critical than others for folding and function. As a result, several authors have argued that site-specific effects are also important to incorporate in molecular evolution models (Meyer and Wilke 2015; Hermans et al. 2025). Moreover, predicting the impacts of amino acid substitutions at particular sites in proteins (known as variant effect prediction) is an active area of research (Bromberg et al. 2024). There is naturally great interest in identifying which non-synonymous mutations are most likely to cause disease or modulate other phenotypes of interest. Methods developed for this purpose are generally of two classes: (1) sequence-based, which rely on deep alignments (Ng and Henikoff 2003) or protein language models (Rives et al. 2021; Elnaggar et al. 2022); (2) structure-based, which attempt to model protein folding and biophysics based on known or predicted structures (Gerasimavicius et al. 2025). In either case, benchmarking these approaches against simple amino acid distance measures, which are not site-specific, is important to assess their relative performance.

Here, we explore a range of existing amino acid distance measures, which have been understudied for molecular evolution applications. We also develop a new experimental exchangeability measure, DEX, which is a consensus of a separate measure we computed based on recent deep mutational scanning data combined with the EX measure. We evaluate the relative utility of all measures for modelling codon substitution patterns and predicting allele frequencies across highly divergent lineages. Our work provides a detailed comparison of the performance of existing measures and points a way forward for molecular evolution models to incorporate more informative amino acid distance measures.

## Methods

### Existing amino acid dissimilarity measures

There are many approaches for capturing pairwise dissimilarity between amino acids, which have been aggregated into several databases and packages. Below, we briefly describe the dissimilarity measures we analyzed and where these values were acquired. Some of these measures represent similarity rather than dissimilarity: we took the complement of all measures as needed (after min-max scaling, see below) so that they all represent similarity or dissimilarity, depending on the analysis. For all asymmetric measures, we took the mean of bidirectional distances between amino acids. Similarity measures were divided by the max similarity, to convert the max value to 1.0.

As mentioned above, the most well-known amino acid dissimilarity measures are Grantham’s distance (Grantham 1974) and Miyata’s distance (Miyata et al. 1979), which were partially motivated by two earlier measures. The first was Sneath’s D (Sneath 1966), which is based on 134 physiochemical characteristics of amino acids. The D index represents the percentage of these characteristics that are not shared between two amino acids. The second earlier measure is Epstein’s coefficient of difference (Epstein 1967), based on polarity and size. We parsed these four measures, as well as the EMPAR measure (Rao 1987), which is based on topological and physicochemical features, from the PARD python package (Lhotte and Taupin 2022). We manually parsed an additional dissimilarity measure, “Xia”, that captured which amino acids neighbour each other in sequences (Xia and Xie 2002). These authors reported their dissimilarity matrix as the average of their neighbour-based approach and Miyata’s distance, which we multiplied by two and from which we removed Miyata’s distance to back-calculate the neighbour score. A final existing matrix of pairwise amino acid (dis)similarities we parsed is the Conformational Similarity Weight (CSW) matrix (Kolaskar and Kulkarni-Kale 1992), which represents similarities based on pairwise comparisons of observed distributions of backbone dihedral angles (ϕ and ψ) for amino acids across the crystal structures of 102 proteins. Because the CSW matrix contains zero-valued entries, which would mean substitutions between certain amino acids are impossible, we replaced these values with a small positive value (0.01 × the matrix maximum, *i.e*., 0.1).

A different approach for computing amino acid dissimilarities is to summarize the impacts that substitutions have on protein function, based on datasets where substitutions in proteins have been experimentally investigated. This was the approach taken to develop the experimental exchangeability (EX) measure, that was based on experimental substitutions across 12 proteins (Yampolsky and Stoltzfus 2005). This measure, which we also acquired from PARD, was estimated based on fitting a power law model to observed impacts of each amino acid pair, on a variety of phenotypes. A similar measure, DeMaSk (Munro and Singh 2021), was recently developed based on 18 proteins where deep-mutational scanning has been performed, meaning nearly all possible amino acid substitutions were tested. These authors used an approach that involved transforming the experimentally obtained substitution impacts into rankings per site, to estimate the overall relative exchangeability per amino acid pair. The new dissimilarity measure we computed (DMS-EX) is closely related to these two prior measures but differs in the experimental datasets and transformation approach used, as described below.

Amino acid substitution matrices, such as BLOSUM62 (Henikoff and Henikoff 1992) and VTML200 (Müller et al. 2002), are well-known representations of pairwise substitution rates between amino acids, which share similarities with the above measures. The BLOSUM62 matrix is based on empirical differences between protein sequences with <= 62% identity, while VTML200 is a similar matrix but based on a maximum likelihood model of evolutionary divergence. Amino acids with high substitution rates in such matrices are generally more structurally similar and are therefore likely to have less impact on protein structure and function. However, such empirical matrices do not disentangle selective effects from mutational biases, which will also influence pairwise substitution rates between amino acids. Thus, we included BLOSUM62 and VTML200 as representative substitution matrices for comparison with other measures, but it would be inappropriate to use them to represent amino acid similarity in codon substitution models, where mutational biases are calculated separately. We converted the log-odds scores to probabilities for our analyses.

Rather than estimating continuous differences, amino acid substitutions are often binned as conservative and radical, based on physicochemical groupings (Zuckerkandl and Pauling 1965; Hughes et al. 1990). We included these approaches in our molecular evolution models to assess whether continuous measures or binary classifications result in better model fits. We considered two distinct groupings (“Zhang RvC” and “Weber RvC”) based on polarity and volume (Zhang 2000; Weber and Whelan 2019), as well as groupings based on charge (Zhang 2000). To include these groupings in comparable analyses, we set amino acid pairs as having similarities of 99% or 1%, for conservative and radical substitution pairs, respectively.

The above measures are representative of how amino acid dissimilarity is typically captured in bioinformatic analyses. However, diverse amino acid characteristics, often based on dimension reduction approaches applied to many physicochemical properties, have been presented in the literature, which we also incorporated into our analyses. These approaches represent amino acid characteristics rather than proposed dissimilarity measures, as described below. We computed dissimilarity measures from each of these sets of characteristics by min-max scaling each individual variable (*i.e.,* scaled them to have a range between 0 and 1): $x_{i}^{\prime }=\frac{x_{i}-x_{min}}{x_{max}-x_{\min}}$.

Where $x_{\min}$ and $x_{\max}$ are the minimum and maximum values for a variable across all amino acids, and $x_{i}$ is the value for amino acid i. The Euclidean distance was then computed between amino acids based on all variables per measure: $d_{i,j}=\sqrt{\sum_{k=1}^{n}{\left(x_{i,k}^{\prime }-x_{j,k}^{\prime }\right)}^{2}}$, where $d_{i,j}$ is the distance between amino acids i and j, and n is the total number of variables. From the set of metrics aggregated in the Peptides R package v2.4.6 (Osorio et al. 2015), we parsed: (1) “Cruciani”: three scaled principal component scores (Cruciani et al. 2004); (2) “FASAGI”: six components representing diverse amino acid characteristics (Liang and Li 2007); (3) “Kidera”: ten orthogonal factors based on multivariate analysis (Kidera et al. 1985); (4) “zScales”: five factors representing physicochemical observations, including based on thin-layer chromatography and nuclear magnetic resonance data (Sandberg et al. 1998); (5) “VHSE”: 8 principal components based on hydrophobic, steric, and electronic properties (Mei et al. 2005). We similarly defined the “Atchley” measure based on five scaled factors previously published to summarize 54 amino acid features (Atchley et al. 2005).

Similar to the above methods which performed dimension reduction across amino acid characteristics, we repeated an analogous approach based on amino acid characteristics across different functional groupings in the AAontology database (Breimann et al. 2024). This database provides an ontology of amino acid scales, largely based on the AAindex database (Nakai et al. 1988; Tomii and Kanehisa 1996; Kawashima and Kanehisa 2000; Kawashima et al. 2008), which itself parsed amino acid characteristics from over 100 articles. The AAontology (“AA-Ont.”) database includes 586 amino acid scales split across eight categories: composition (“Comp.”), conformation (“Conf.”), energy, others, polarity (“Pol.”), shape, structure/activity (“Struc.”), and volume (“Vol.”). We computed principal coordinates analyses across all scales per category and computed a single dissimilarity measure per category by performing a similar calculation as above. Rather than min-max scaling, we retained the original values and instead weighted the difference in each principal component by the variance explained: $e_{i,j}=\sqrt{\sum_{k=1}^{n}p_{k}{\left(x_{i,k}-x_{j,k}\right)}^{2}}$, where $e_{i,j}$ is the distance between amino acids i and j,n is the total number of principal components included, and $p_{k}$ is the proportion of variance explained by component k. We used this same approach to generate a single dissimilarity measure (“Venkatarajan”) from another analysis that provided five factors (and corresponding eigenvalues) representing 237 physicochemical properties (Venkatarajan and Braun 2001).

To compare all these measures visually, we computed the mean exchangeabilities per amino acid for each measure. These exchangeability values were conceptually based on Graur’s stability index (Graur 1985), but based on amino acid similarities rather than distances. Specifically, we computed the mean amino acid similarity based on every possible single non-synonymous mutation in a codon. We visualized these results in a clustered heatmap based on taking the standard score of the exchangeabilities per measure and then computing Euclidean distances for both amino acids and measures (*i.e.,* for rows and columns separately) and then performing hierarchical clustering with the complete method. We also ran Principal Component Analyses (PCAs) on the exchangeabilities scaled per measure to visualize the relative similarity of measures. We built one PCA based on all measures and one PCA with these major outlier measures removed, for better visualization: CSW, EMPAR, Comp. (AA-Ont.), Conf. (AA-Ont.), RvC (charge), RvC (Zhang), and Venkatarajan.

### Re-calculating classic distance measures

In addition to the standard Grantham’s distance values, we re-calculated Grantham’s distance based on the composition, polarity, and molecular volume values presented in the original publication (Grantham 1974). We did so because we noticed rounding errors in the presented distance values. We calculated the raw Grantham’s distance between amino acids i and j as: $g_{ij}=\sqrt{{\left(\frac{c_{i}-c_{j}}{\mu_{\Delta c}}\right)}^{2}+{\left(\frac{p_{i}-p_{j}}{\mu_{\Delta p}}\right)}^{2}+{\left(\frac{v_{i}-v_{j}}{\mu_{\Delta v}}\right)}^{2}}$, where c,p, and v represent composition, polarity, and volume, and $\mu_{\Delta c},\;\mu_{\Delta p}$, and $\mu_{\Delta v}$ represent the mean absolute difference between all amino acid pairs based on each variable alone ($\mu_{\Delta c}=0.7393684,\;\mu_{\Delta p}=3.134211$, and $\mu_{\Delta v}=50.06053$). We then calculated the scaled Grantham’s distances, $G_{ij}$, which are scaled so that the mean distance across all pairs is 100, following from the original calculation: $G_{ij}=g_{ij}\times \left(100/\mu_{d}\right)$, where $\mu_{d}$ is the mean raw Grantham’s distance between all amino acid pairs ($\mu_{d}=1.968261$). These final scaled Grantham’s distances are similar to the originally published distances (mean difference in values: 0.03), but there are some noteworthy differences, including a maximum absolute difference of 9.83 units between the original and re-calculated distances (for the distance between aspartic acid and tryptophan, which is under-estimated in the original table).

Grantham’s distance is the most commonly used, general-purpose, amino acid distance measure, *e.g*., (Graur 1985; Chowell et al. 2019). However, substantial work has been conducted to further refine estimates of amino acid properties since it was originally published. Accordingly, we also re-calculated Grantham’s distances with updated volume and polarity estimates. We did not adjust the composition estimates as these were specifically defined for the original measure. To this end, we used more recently computed amino acid volumes that have been shown to have lower variance compared to other approaches (Tsai and Gerstein 2002). For a measure of polarity we followed the same approach of scaling and then averaging the polar requirement (Woese et al. 1966) and a measure of hydrophobicity per amino acid. We used an estimated measure of polar requirement based on molecular dynamics simulations, rather than the original values, as the former avoids systematic experimental error (Mathew and Luthey-Schulten 2008). In addition, rather than the hydrophobicity measure that was originally used (Aboderin 1971), we used a measure that has been shown to perform best (out of 98 hydrophobicity measures) for distinguishing peptides based on hydrophobicity features (Naderi-Manesh et al. 2001; Simm et al. 2016). We then multiplied the hydrophobicity metric by −1 (to make the two variables positively correlated) and performed min-max normalization of the two measures across the amino acids (so that they each range from 0 to 1). We then computed the mean of these scaled values to represent each amino acid’s polarity.

We used these same steps to re-calculate Miyata’s distances based on these updated hydrophobicity and volume measurements. Raw Miyata’s distances were calculated following from the original approach (Miyata et al. 1979): $m_{ij}=\sqrt{{\left(\frac{p_{i}-p_{j}}{\sigma_{\Delta p}}\right)}^{2}+{\left(\frac{v_{i}-v_{j}}{\sigma_{\Delta v}}\right)}^{2}}$, where $\sigma_{\Delta p}$ and $\sigma_{\Delta v}$ are the standard deviations in pairwise differences in polarity and volume between all amino acids. We conducted a supplementary comparison of these varied computations of Grantham’s and Miyata’s distances, to ensure that our conclusions regarding these well-known measures did not depend on minor differences in how they are computed.

### Computing a new experimental exchangeability measure

We downloaded all 217 deep mutational scanning datasets from the ProteinGym database v1.1 (Notin et al. 2023). We then strictly filtered these datasets down to a smaller high-quality and independent set. This involved first retaining only datasets with at least 95% of sites tested (minimum of 20) and a mean of at least 15 (out of 19) amino acids tested per site. We then subset the target sequences that were used for the experiments to be only residues where all 19 replacement amino acids were experimentally tested (with multi-substitutions excluded). We also dereplicated datasets with near-identical proteins by clustering all target protein sequences with CD-HIT v4.8.1 (Fu et al. 2012) at 70% identity and keeping only the representative sequence for each cluster. We also determined how each score was transformed based on the original published articles and excluded scores with ambiguous or unclear transformations. At this point, 79 datasets remained for which we could unambiguously determine what the score represented in terms of the transformations employed. These included 60 datasets from the same study (Tsuboyama et al. 2023). We decided to keep only one dataset per independent publication to help avoid biases and to include substitution impacts across more varied proteins and experimental protocols, which resulted in a final set of 18 deep mutational scanning datasets (Supplementary Table 1) (Melnikov et al. 2014; Klesmith et al. 2015; Majithia et al. 2016; Weile et al. 2017; Wrenbeck et al. 2017; Lee et al. 2018; Mighell et al. 2018; Soh et al. 2019; Chen et al. 2020; Jia et al. 2021; Chen et al. 2023; MacRae et al. 2023; Suphatrakul et al. 2023; Tsuboyama et al. 2023; Weeks and Ostermeier 2023; Clausen et al. 2024; Estevam et al. 2024; Vanella et al. 2024). For datasets representing raw scores we set a minimum score of 0.01. We then transformed all the scores across these datasets to a natural log scale.

Following the approach developed to generate the EX measure (Yampolsky and Stoltzfus 2005), we treated substitution scores separately depending on whether they were derived from buried or exposed regions of the proteins. To identify these regions, we analyzed all AlphaFold2 structures (Akdel et al. 2022) associated with the target protein sequences. We first ran mkdssp v3.0.0 (Touw et al. 2015) to identify secondary structure features, including solvent accessibility (Kabsch and Sander 1983). We then calculated the relative solvent accessibility per residue using previously determined maximum allowed solvent accessibilities per amino acid and a Python script to conduct this calculation (Tien et al. 2013). We defined buried sites as those with a relative accessible surface area below 0.2, with all others defined as exposed. We used the same workflow to compute the relative accessibility of all amino acids across the AlphaFold-predicted structures across the entire *Escherichia coli*, human and yeast proteomes (v4) (Akdel et al. 2022). We then calculated the mean proportion at which each amino acid is found within buried and exposed sites, for each species separately. We then took the mean of these three mean values per amino acid to get an overall estimate of the background proportion of cases that each amino acid is found at buried vs. exposed sites.

To calculate our custom deep-mutational scanning exchangeability score, we considered all residues in the 18 target proteins with at least 15 amino acid substitutions tested. For each amino acid substitution type, and within each tested protein and site-type (buried or exposed), we computed robust Z-scores. Specifically, for all exposed (e) residues, we computed the robust Z-score (r) for a given substitution (j→k) at site i in protein p as: $r_{j\to k,i,p,e}=\frac{x_{j\to k,i,p,e}\;-\;x_{\mathrm{median},p,e}}{1.4826\;\times \;x_{\mathrm{MAD},p,e}}$. Where $x_{\mathrm{median},p,e}$ is the median score for all substitutions at exposed residues in this protein and $x_{MAD,p,e}$ is the median absolute deviation of all scores at exposed sites. The median absolute deviation is multiplied by 1.4826 to approximate the standard deviation when the underlying data is normally distributed (Rousseeuw and Leroy 1987). We then computed the median robust Z-score per amino acid substitution type, per protein, and for buried and exposed sites separately, and then computed the median of these values across all genes, meaning that for each amino acid substitution type there is a score for the buried and exposed sites separately ($r_{j\to k,b}$ and $r_{j\to k,e}$). Finally, we computed a weighted score per substitution type: $s_{j\to k}=r_{j\to k,b}\times b_{j,b}+r_{j\to k,e}\times b_{j,e}$, where $b_{j,b}$ and $b_{j,e}$ are the background frequencies of the amino acid j across the background proteomes at buried and exposed sites, respectively. We refer to this custom measure as DMS-EX.

We also computed consensus measures based on subgroups of measures using the DISTATIS approach (Abdi et al. 2012) with the DistatisR package v1.1.1. DISTATIS yields compromise factor scores (analogous to PCA scores) based on multiple distance matrices. In this case, the factor scores capture where amino acids are positioned in the compromise space, weighted by the eigenvalues. We retained all factors with positive eigenvalues and computed a consensus measure for each grouping of measures by computing the Euclidean distances between pairwise amino acids based on these factors.

### Fitting codon substitution models to diverse alignments

We fitted codon substitution models on separate gene alignments across 6 *Drosophila*, 190 mammal, and 71 *Streptococcus* species, to ensure that a diverse set of independent lineages was evaluated. *Drosophila* and mammal alignments were chosen given that these lineages are well-studied and have resources available. We acquired the previously prepared alignments and species trees for these lineages from flyDIVaS v1.2 (Stanley and Kulathinal 2016), for the six species in the “melanogaster species group”, and from orthoMaM v12 for the mammalian data (Allio et al. 2024).

We generated custom alignments of the *Streptococcus* species. We selected this genus as a representative of prokaryotes which has high species diversity among sequenced isolates. We downloaded 3,270 genomes from GenBank and then identified 71 different *Streptococcus* species defined at a 95% average nucleotide identity using skani v0.2.2 (Shaw and Yu 2023). We selected one genome for each of these species clusters and ran Bakta v1.8.1 (Schwengers et al. 2021) followed by PanTA v1.01 (Le et al. 2024) to produce a pangenome breakdown across the genus, and identified all gene families found in at least 68/71 genomes. We then ran MACSE2 (Ranwez et al. 2018), specifically the OMM_MACSE v12.01 workflow, to build codon alignments for each of these gene families across these genomes and built trees for each gene family with fasttree v2.1.11 using default settings (Price et al. 2010).

For the three lineages, we decided to concatenate genes together to ensure sufficient data for model fitting. This was instead of running codon models on every individual gene, which would have limited numbers of substitutions present for fitting robust models. Before doing so we estimated the numbers of synonymous and non-synonymous substitutions across all gene alignments across all three lineages using Fitch parsimony (Fitch 1971). We retained only alignments with a length between 200–1000 codons, inclusively, and with at least 20 synonymous and 20 non-synonymous substitutions. We then identified 20 non-overlapping, random sets of 12 genes for each lineage and concatenated these 12 gene alignments together for each random set. Only genomes with all 12 genes present were included within each set. We generated new trees per alignment set with fasttree for the mammalian and *Streptococcus* concatenated alignments.

To fit the substitution models, we ran CODEML from the PAML package v4.10.7 (Yang 2007), as wrapped in the Biopython Python package v1.85 (Cock et al. 2009). We ran CODEML on all alignments (described below) with both the M0 model (standard d_N_/d_S_) and with an amino acid distance matrix corresponding to the measures described above, which was specified with the ‘aaDist’ option (Goldman and Yang 1994). We compared Akaike Information Criterion (AIC) values across the fitted models ranked from lowest (best) to highest. We ran the first 10 sets of alignments on all individual measures and used these to identify the best-performing measures, which we then used as the basis for the consensus amino acid measures produced with DISTATIS, as described above. We evaluated these consensus measures on the remaining 10 sets of alignments.

### Segregating non-synonymous variants across humans and *E. coli*

We acquired 2,114 previously generated protein-coding genes across strains of *E. coli* (Vigué et al. 2022), which are available in this Zenodo repository: https://zenodo.org/records/5774192. There is a range of 60,002–61,145 strains per gene in this dataset. We translated the DNA alignment to amino acids and then re-aligned these sequences with MUSCLE v5.1 (Edgar 2004), ran in super5 mode for speed. We then used custom Python scripts to determine the consensus amino acid and codons at each site in the alignments. For the consensus codon, we took the most frequent codon per site that matched the consensus amino acid. We only considered sites that contained unambiguous amino acid residues for at least 60,000 strains in the alignment.

We similarly downloaded and parsed segregating non-synonymous mutations across human genotypes. We downloaded all exonic VCFs (*i.e.,* for all chromosomes) from the gnomAD variation database (v4.01) (Karczewski et al. 2020). These variant calls include annotations of non-synonymous calls across all proteins. We downloaded protein and transcript sequences from GENCODE v45 (Frankish et al. 2023), and identified the proteins for which RaSP predictions were available. We then used a custom Python script to parse out non-synonymous variants across all chromosomes, including information on the amino acid residue in the protein, the amino acid change, and the frequency. The remaining steps were the same as the *E. coli* analysis above, where the GENCODE protein (and codon) sequences were used as the references for predicting site-specific alleles. When performing correlations between minor allele frequency and exchangeability, we corrected for multiple tests using the Benjamini-Hochberg procedure (Benjamini and Hochberg 1995).

### Running site-specific effect predictions

To provide context for how informative amino acid dissimilarity measures perform at predicting deleterious amino acid substitutions compared to state-of-the-art methods, we also analyzed the outputs from two deep learning approaches. These include RaSP (Blaabjerg et al. 2023) for protein structure-based predictions and VespaG (Marquet et al. 2024) as a method based on embeddings from protein language models. These approaches are representative of the two main approaches used for predicting substitution effects: structure and sequence-based methods. Structure-based approaches are particularly computationally intensive to run, thus we opted to use pre-existing predictions based on predicted structures across the human proteome (https://doi.org/10.17894/ucph.7f82bee1-b6ed-4660-8616-96482372e736). Unfortunately, no such structural predictions were available for *E. coli* to our knowledge. We ran VespaG (commit: 660b17c6964eb6db8e3f3bee2b8bbd3f9f574d23) with the ESM-2 protein language model embeddings (Lin et al. 2023).

### Visualization and working environments

Commands were run using the R (v4.2.2) and Python (v3.10) programming languages. Plots were generated using the R packages ggplot2 v3.5.1 (Wickham 2016) and ComplexHeatmap v2.20.0 (Gu 2022). We installed command-line tools from the Bioconda project (Grüning et al. 2018), when possible, and parallelized commands using GNU Parallel version 20240222 (Tange 2011). We used Claude AI to help check for errors across our scripts and manuscript.

## Results

### Exploring existing amino acid distance metrics and introducing a new measure

Many different measures capturing amino acid distances have previously been proposed (Table 1; Supplementary Table 2). These include binary measures of radical vs. conservative substitutions, as well as many scales based on physicochemical properties. A more recent set of approaches is based on experimentally observed impacts of amino acid substitutions in proteins (Yampolsky and Stoltzfus 2005; Munro and Singh 2021). Partially motivated by these approaches, we computed a new measure, DMS-EX, which is based on publicly available deep mutational scanning datasets (Notin et al. 2023). DMS-EX differs from past approaches in terms of the exact datasets included and especially because it incorporates the magnitude of impact differences, which are all on the same scale across datasets, into the measure, rather than just the relative rankings (see Methods).

**Figure 1:**
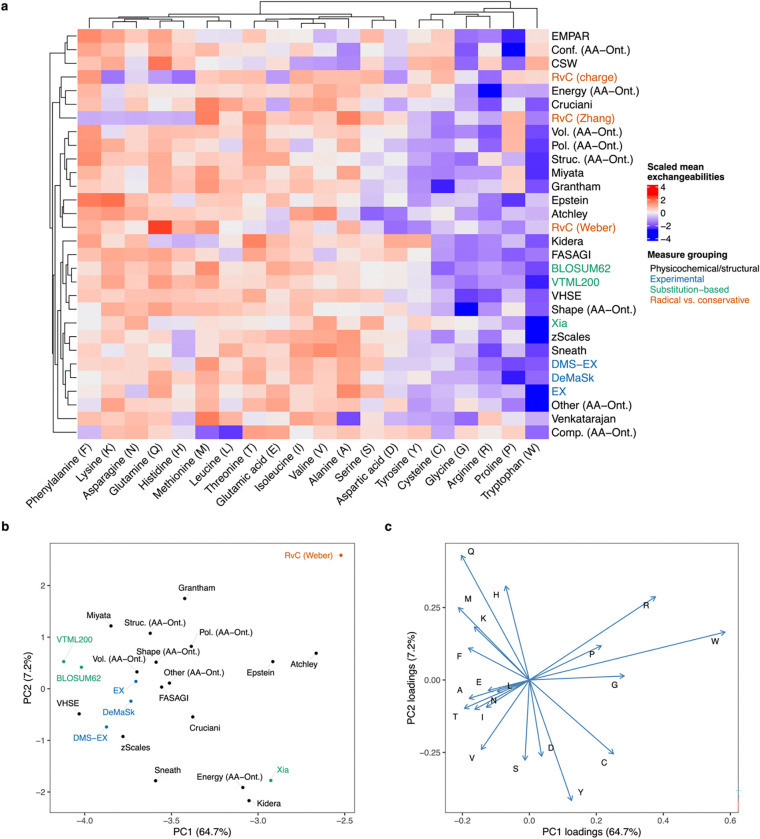
Comparison of amino acid (dis)similarity measures. (a) Mean exchangeabilities per measure, averaged across all codons for each amino acid. A codon’s mean exchangeability was calculated as the mean amino acid similarity between all amino acids that could arise from a single non-synonymous mutation to the codon. Rows represent the different metrics analysed (which were all converted to similarities, see the main text for descriptions). Scaled values were hierarchically clustered based on Euclidean distances using the complete method, for both the rows and columns separately. (b) First two principal components (PCs) of Principal Components Analysis (PCA) of all dis(similarity) measures based on Euclidean distances. Outlier measures were excluded from this PCA for better visualization (see text and Supplementary Figure 1). (c) Same PCA as in panel b but displaying the loadings that each amino acid contributes to the separation. Percentage of variance explained by each PC is indicated in parentheses.

To visualize the similarity across existing measures, in addition to our novel measure, DMS-EX, we performed a comparison of 30 differing measures. For each measure we computed the exchangeability per amino acid, which represents the mean similarity to all other amino acids that can be reached by one non-synonymous mutation. We then clustered these exchangeability values to visualize variation across measures (Figure 1a). At a broad level, the measures are consistent with one another in terms of dividing amino acids like tryptophan and arginine (mean standard scores < −1.1) with low exchangeability from others with higher exchangeability, such as glutamine and methionine (mean standard scores > 0.65). As expected, the measures are correlated to one another on average (median Spearman’s ρ = 0.45; standard deviation: 0.27), and we observed groupings between similar measures, such as DMS-EX with EX and DeMaSk, which are all based on experimental data. However, certain measures are major outliers driving the variation (see Methods; Supplementary Figure 1). Excluding these outliers, we visualized the relative similarity in these measures based on the first two principal components (Figure 1b). A single component explains a substantial amount of the variance (64.7%), which is consistent with clustering driven by two main types of approaches: those based on physicochemical measures (*e.g*., RvC and Grantham) and those based on empirical substitutions (*e.g*., BLOSUM62 or EX). We observed that a small number of amino acids display an outsized impact on the variance across measures, particularly tryptophan and arginine, rather than an overall shift in exchangeability across all amino acids (Figure 1c).

### Fitting models of protein-coding gene evolution with amino acid distance measures

To assess how well these amino acid distance measures explain actual non-synonymous substitutions patterns, we applied a previously developed codon substitution model that incorporates an amino acid distance matrix (Goldman and Yang 1994). We applied this model to diverse alignments representing 71 *Streptococcus* species, 6 *Drosophila* species, and 190 mammalian species. We constructed 10 alignments of 12 concatenated genes randomly sampled (non-overlapping) for each of these independent lineages and applied the codon substitution model for each amino acid distance matrix to each separate alignment.

**Figure 2:**
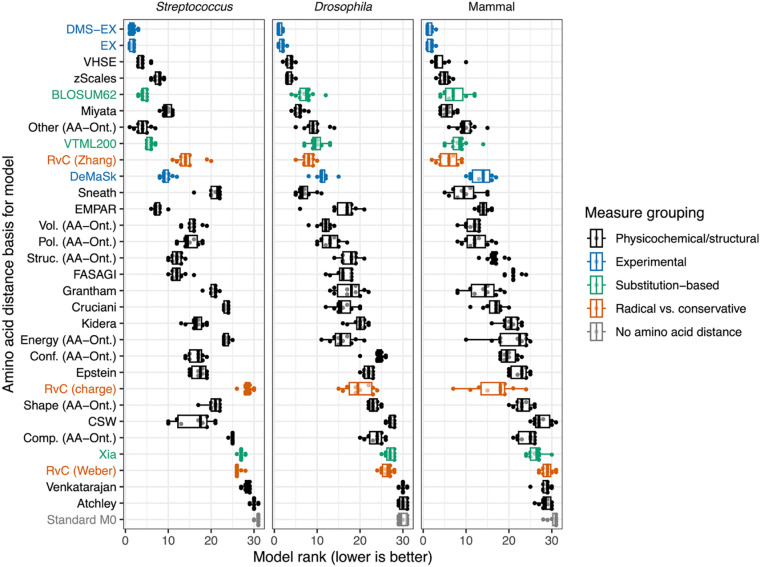
Comparison of model rankings, based on Akaike Information Criterion values, for models applied to 10 different protein-coding gene alignments (with each alignment containing 12 randomly selected concatenated genes) across three highly diverged lineages using different amino acid similarity metrics.

Based on the rankings of model performances across these alignments (Figure 2), all models that incorporated amino acid distances performed better than the standard codon substitution model (*i.e.*, the M0 model in PAML, a simple molecular model of non-synonymous vs. synonymous substitutions) that excludes these distances. Our custom measure, DMS-EX (mean rank: 1.5) and the EX measure (mean rank: 1.7) performed best on average across all three lineages. The difference in Akaike Information Criterion values in models based on these two measures was not significantly different from 0 (Wilcoxon test, *P*=0.37), indicating that these measures perform similarly well. In addition, because Grantham’s and Miyata’s distances are so well known in molecular evolution, we updated these measures since more accurate estimates of the volume, polarity and hydrophobicity of amino acids are now available (see Methods). Interestingly, these updated values provided no clear improvement over the original measures (Supplementary Figure 2).

**Figure 3:**
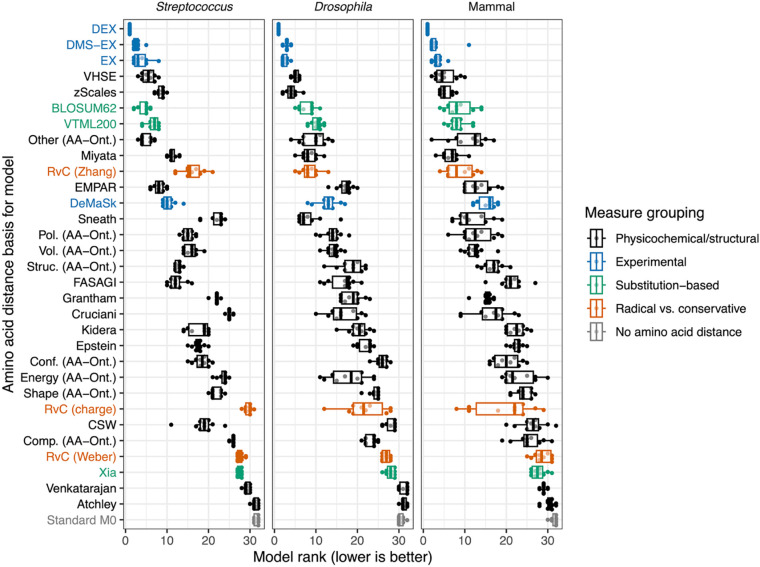
Comparison of model rankings, based on Akaike Information Criterion values, for models applied to 10 different protein-coding alignments across three highly diverged lineages but with different amino acid similarity measures. This result corresponds to an independent set of codon alignments compared to the earlier analysis used to identify the top measures to test in new combinations.

We reasoned that a consensus measure based on several top-performing measures should perform best for modelling non-synonymous substitution patterns. To this end, we generated numerous consensus measures with the DISTATIS approach (see Methods), based on different combinations of the top six performing measures (excluding BLOSUM62 since it incorporates mutational biases). We also evaluated consensus measures incorporating DeMaSk, because this measure was constructed using a very similar approach to the one we used to generate DMS-EX. We evaluated these consensus metrics on a new set of 10 alignments for each of the three lineages (Supplementary Figure 3). We found that the separation between the top measures was less clear, but the consensus measure that combined our DMS-EX approach and EX was ranked best on average (mean rank: 3.2). We refer to this new consensus measure as DEX, standing for “DISTATIS-based consensus of experimental exchangeability”. Interestingly, we noted some instances of major outliers, particularly the model based on the combined EX and DeMaSk measures, which performed best for the *Streptococcus* alignments specifically (mean rank: 1.6) but did not perform well for the mammal and *Drosophila* alignments (mean rank: 5.7). More generally, we found that DEX fit best across all alignments compared to the individual measures based on model rank (Figure 3). In addition, the difference in Akaike Information Criterion values for models fit on DEX vs. DMS-EX (as the next best-performing measure) were significantly greater than 0 (median difference: 164.5; Wilcoxon test *P* < 0.001).

**Figure 4:**
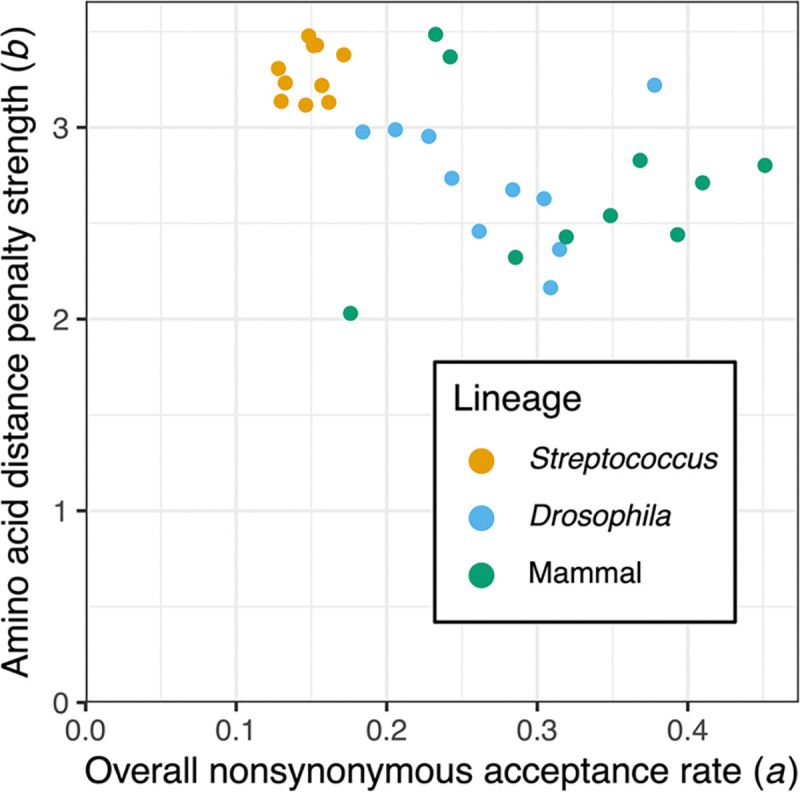
Comparison of parameters inferred in codon substitution models across three divergent lineages. These results are specifically for models using the DEX amino acid distances. The two parameters, *a* and *b,* are inferred within a codon substitution model that incorporates amino acid distances, but which is no longer commonly used.

Although not the primary goal of this study, we were also interested in testing whether incorporating amino acid distances into codon substitution models would improve the inference of selection efficacy across lineages. To do so, we ran an early implementation of a codon substitution model in PAML (Goldman and Yang 1994), which does incorporate amino acid distances and which is no longer commonly used. Standard d_N_/d_S_ codon substitution models (*i.e*., those that do not incorporate amino acid distances) infer one or more parameters representing the ratio of non-synonymous to synonymous substitution rates, which are captured by the parameter *a* in the models we ran. A benefit of applying this codon substitution model that incorporates amino acid distances is that an additional parameter (*b*) is also inferred, which represents the extent to which substitutions between dissimilar amino acids are penalized. As discussed below, we hypothesize that this parameter could reflect differential selective efficacy, driven by differences in effective population size. Indeed, we observed a qualitative shift between these inferred parameters between the *Streptococcus* alignments and the other alignments (Figure 4). In particular, there is an apparent negative association between parameters *b* and *a*, which is partially driven by differences between the lineages (Kruskal-Wallis rank sum tests *P* < 0.01). This result is consistent with more effective (or stronger) selection against substitutions between dissimilar amino acids in bacteria compared to eukaryotes, which is also associated with a lower relative rate of non-synonymous substitutions in general in bacteria. These results support the idea that incorporating continuous amino acid distances into codon substitution models can improve the prediction of selection exicacy, although the interpretation of the data may not be straightforward (see Discussion).

### Amino acid polymorphism frequencies are associated with predicted impacts

Although we were primarily interested in evaluating these measures in the context of codon substitution models, amino acid distance measures are relevant to many other bioinformatics areas. This is particularly true for tools that predict the fitness impact of individual amino acid substitutions, which usually focus on segregating mutations within species. Thus, we decided to also evaluate how well these differing amino acid measures capture deleterious mutations across datasets of segregating non-synonymous variants.

Due to the action of purifying selection, deleterious mutations are less likely to rise to high frequencies in populations. We evaluated how well the pairwise amino acid distances across all the measures correlated with the mean frequencies of each type of amino acid substitution across *E. coli* and human individuals. We chose these two highly divergent lineages to once again ensure that our results were generalizable to diverse lineages. We analyzed 2,114 proteins across an alignment of over 61,145 *E. coli* strains that was generated in a previous study (Vigué et al. 2022). We identified 100,360 segregating replacement mutations across these proteins and computed the mean allele frequency for each pair of amino acids per substitution. We performed a similar analysis across 2,077,326 non-synonymous variants across the gnomAD human variation database (with a range of 100,062 – 1,461,894 samples). In addition to the two amino acid distance measures, we also included two Deep Learning approaches designed specifically for inferring the site-specific impacts of non-synonymous mutations: RaSP and VespaG.

**Figure 5:**
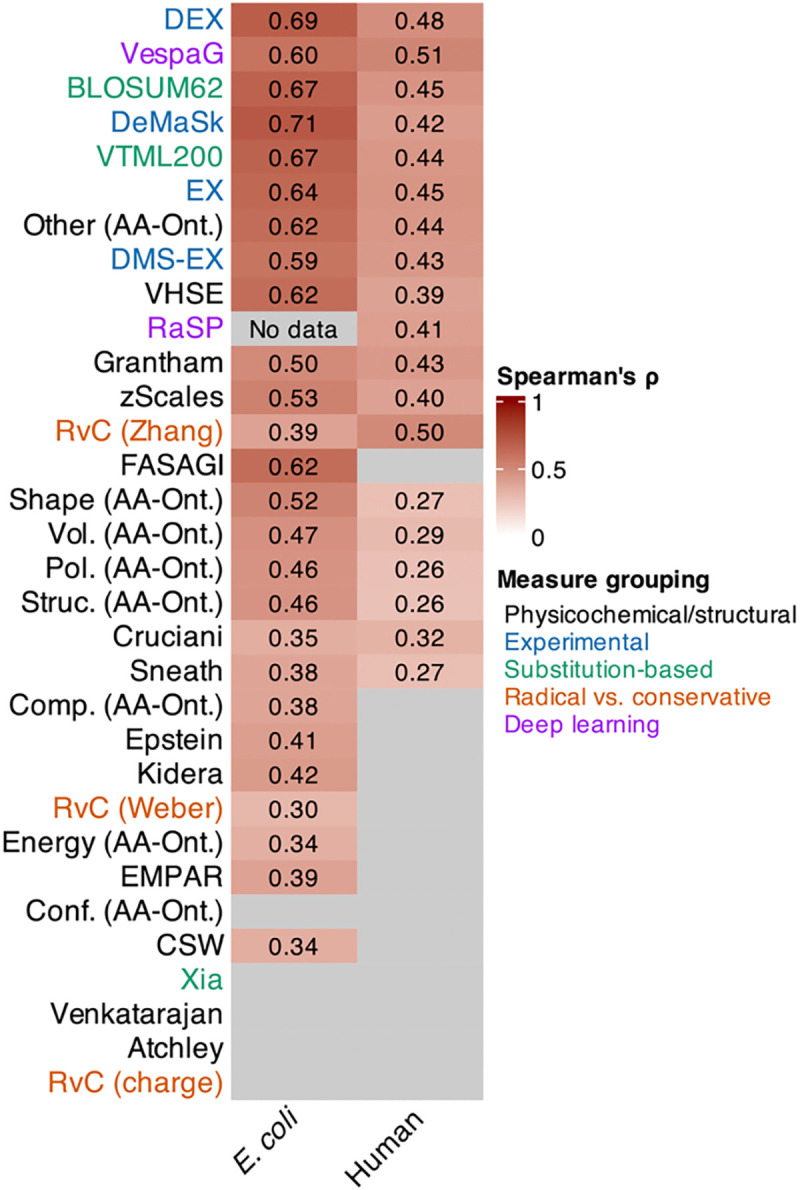
Spearman correlation coefficients (ρ) between mean frequency of segregating non-synonymous mutations and the exchangeability of amino acid pairs. Metrics are sorted by the mean scaled correlation coefficients across both columns. Grey cells indicate non-significant Spearman correlation tests (corrected P-value >=0.05), or no data available (for RaSP in *E. coli*).

As expected, we identified a positive association between the exchangeability of amino acid pairs and the mean frequency of these substitutions (Figure 5). This signal was stronger across *E. coli* strains compared to humans. This overall observation is consistent with substitutions between more dissimilar amino acids being more deleterious and thus these substitutions being less likely to spread throughout the population. As before, our primary interest here was in the relative rankings of these measures’ correlations. These rankings differed substantially from the ranking of the measures evaluated in the codon substitution models. In *E. coli*, DeMaSk was most correlated (Spearman’s ρ=0.707; adjusted *P* < 0.001), followed by DEX (Spearman’s ρ=0.688; adjusted *P* < 0.001). In contrast, across humans, VespaG performed best (Spearman’s ρ=0.515; adjusted *P* < 0.001), followed by RvC (Zhang; Spearman’s ρ=0.497, adjusted *P* < 0.001) and DEX (Spearman’s ρ=0.477; adjusted *P* < 0.001). Nevertheless, by assessing the performance of these approaches jointly across these two highly diverged lineages (*E. coli* and humans), our results show that DEX performs best overall (Figure 5).

**Figure 6:**
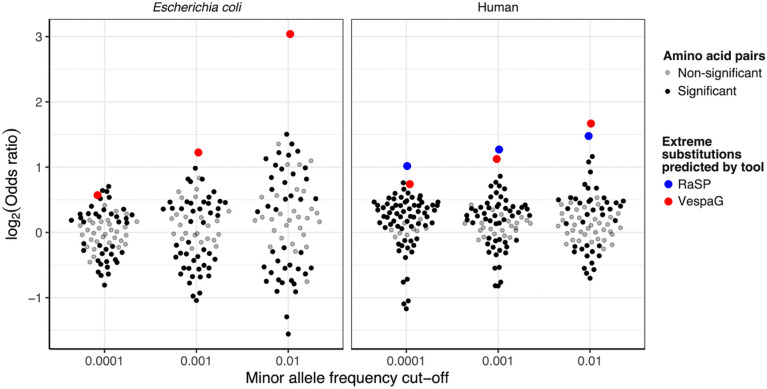
Enrichment of rare segregating replacement mutations among most extreme preference predictions across *E. coli* and humans. Odds ratio of replacement polymorphisms that are below specified frequency cut-offs that are in the lowest 1% quantile of preference predictions (*i.e.*, predicted to be among the 1% most extreme changes) or above this quantile. This was formulated as a 2×2 contingency test for each comparison. Significance based on a Fisher’s exact test (corrected *P* < 0.05) is indicated by black coloured dots.

RaSP and VespaG are two deep learning tools designed for predicting variant effects and are representative of well-performing structure-based and sequence-based site-specific prediction tools, respectively. Although mean VespaG predictions per amino acid pairs were the most correlated in humans, and highly correlated in *E. coli*, with mean frequencies, it is noteworthy that these tools did not perform qualitatively better in this assessment. This result highlights that coarse knowledge about overall amino acid distances provides most information about average impact of substitution types, rather than detailed site-specific information. However, it would be incorrect to infer from this analysis that tools such as VespaG and RaSP are simply comparable to using a general amino acid distance matrix when predicting the impact of individual mutations (as opposed to the mean impact of groups of mutations). We highlighted this point by comparing the enrichment of substitutions among rare vs. frequent mutations (defined at a cut-off of 0.01% of samples) compared across all amino acid substitutions and based on mutations predicted by VespaG and RaSP to be among the 1% most deleterious (Figure 6). Overall, these mutations identified as highly deleterious by VespaG and RaSP are substantially enriched among rare variants compared to what is observed based on amino acid pairs alone. This result emphasizes the ability of these tools to predict the effect of individual mutations on protein sequences, which amino acid distance matrices can only do coarsely.

## Discussion

We developed a new consensus-based experimental exchangeability measure, DEX, and showed that this measure best fits real codon substitution patterns across three diverse lineages. We also explored how diverse amino acid exchangeability measures correlated with mean frequencies of segregating amino acid mutations across populations. We found that the measure rankings dixered in terms of the top-performing measures, but DEX was most consistently among the best measures across all analyses. Both of these general approaches, fitting codon substitution models and comparing to polymorphism frequencies, were previously explored in similar ways when EX was originally presented (Yampolsky and Stoltzfus 2005). Here, we built upon this past work and found that DEX is a particularly performant general-purpose amino acid distance measure. DEX is therefore an improved choice over individual exchangeability-based measures for fitting substitution models, and certainly over measures based on physicochemical properties, such as Grantham’s distance.

Although DEX was most generalizable, it is important to emphasize that no single amino acid distance measure will be most informative across all proteins and lineages (Miyata et al. 1979). For instance, transmembrane proteins have very different amino acid substitution profiles compared to others on average (Mokrab et al. 2010). Similarly, although we found that DEX performed best overall, we also found that a similar consensus measure that included DeMaSk best fit *Streptococcus* gene alignments (Supplementary Figure 3). This could reflect different amino acid preferences on average in this bacterial genus compared to eukaryotes.

This division between the domains was also apparent based on the *a* and *b* parameters that were fit across the focal alignments (Figure 4). Once again, alignments across *Streptococcus* were outliers, in this case by having among the highest values of amino acid penalty strength parameters (*b*) and among the lowest overall non-synonymous acceptance rate parameters fit (*a*). This result is consistent with past observations of varying d_R_/d_C_ across lineages with differing effective population sizes (Weber et al. 2014; Weber and Whelan 2019). In these analyses, some lineages with higher effective population sizes displayed lower d_R_/d_C_ values, which is consistent with more effective selection against radical non-synonymous substitutions (James and Lascoux 2025). The results we observed are also consistent with this explanation, but both our observations and these previous results that focused on d_R_/d_C_ could also be explained by shifting amino acid preferences across lineages. More work is needed to determine how to disentangle shifting amino acid preferences from differential efficacy of selection against deleterious non-synonymous substitutions. The amino acid distance measures we explored herein could serve as a basis for such future explorations.

DEX could also be useful as a static comparison for future variant effect predictors. As we and others have highlighted (James and Lascoux 2025), simply knowing which amino acids are involved in a substitution provides substantial insight into how deleterious it likely will be. However, site-specific context provides additional information (Figure 6), and readers should not mistakenly conclude that static distance measures are equally useful as specialized methods, such as RaSP and VespaG, for predicting amino acid substitution impacts. Nonetheless, when evaluating such tools, it is important to compare to the best possible inference based on amino acid distances alone. Comparing deep learning approaches for predicting substitution impacts to less informative measures, such as Grantham’s or BLOSUM62 distances, would provide misleading confidence in how well a predictor is performing.

In summary, we have compared numerous amino acid distance measures, which largely vary on a continuum from those based on empirical observations to those based on physicochemical properties. DEX is the most generalizable and informative of those we tested. This measure will be useful for future work inferring the efficacy of selection in molecular evolution studies and when benchmarking variant effect predictors.

## Supplementary Material

Supplement 1

## Figures and Tables

**Table 1 -:** Highlighted pairwise amino acid distance measures analyzed in this study

Group	Approach	Description
Radical vs. conservative (RvC)	RvC groupings based on polarity and volume (Zhang 2000)	Classifying substitutions as radical or conservative based on whether amino acids have highly different polarity and volume values (radical substitution) or not (conservative substitution).
Physiochemical/structural	Grantham’s distance (Grantham 1974)	Distance based on values of composition, polarity, and volume for each amino acid.
Substitution-based	BLOSUM62 (Henikoff and Henikoff 1992)	Based on observed substitutions across protein alignments where sequences are <= 62% identity. Confounded with nucleotide mutational biases.
Experimental	Experimental exchangeability (EX) (Yampolsky and Stoltzfus 2005)	Based on a comparison of 9,671 induced amino acid substitutions across 12 proteins.
Experimental	DMS-EX (this study)	New measure of experimental exchangeability that we computed based on deep-mutational scanning datasets.
Experimental	DeMaSk (Munro and Singh 2021)	Similar to DMS-EX and EX but based on a slightly different set of datasets and based on rank-based transformations.
Experimental	DEX: DISTATIS-based consensus of experimental exchangeability (this study)	New measure that we computed as the consensus of EX and DMS-EX, using DISTATIS. This was the top-performing measure across all the matrices that we evaluated.

## Data Availability

Code for running all our analyses is available at this GitHub repository: https://github.com/gavinmdouglas/aa_distance_explore. Key datafiles, including the different amino acid distance and similarity metrics compared herein, are available on Zenodo: https://doi.org/10.5281/zenodo.18927704.

## Significance

Protein-coding genes have long been a focus for researchers studying the strength and direction of selection. By studying non-synonymous substitutions, those that change amino acids, it is possible to estimate the relative strength of selection. Despite widespread interest in such approaches, information on which amino acids are exchanged is underused in molecular evolution models. This is partly because many different measures exist for quantifying amino acid distances, particularly those based on physicochemical properties. A newer class of amino acid distance measures is derived from deep mutational scanning datasets, where virtually every possible substitution is tested for its impact on protein function. We characterised and compared 30 amino acid distance measures, including a novel measure based on deep mutational scanning data. We highlight differences in how well these measures fit real substitution and polymorphism datasets. Overall, we find that DEX, which is a consensus of our new measure and an existing experimental exchangeability measure, represents the best available amino acid distance measure to incorporate into molecular evolution models.
