# Supplementary material for: DEX: a consensus-based amino acid exchangeability measure for improved codon substitution modelling
Source: bioRxiv. 2026 Mar 12:2026.03.09.710665. Preprint. [Version 1] doi: 10.64898/2026.03.09.710665 (PMC13060851; doi:10.64898/2026.03.09.710665)
Supplement: Supplement 1 [file media-1.pdf]

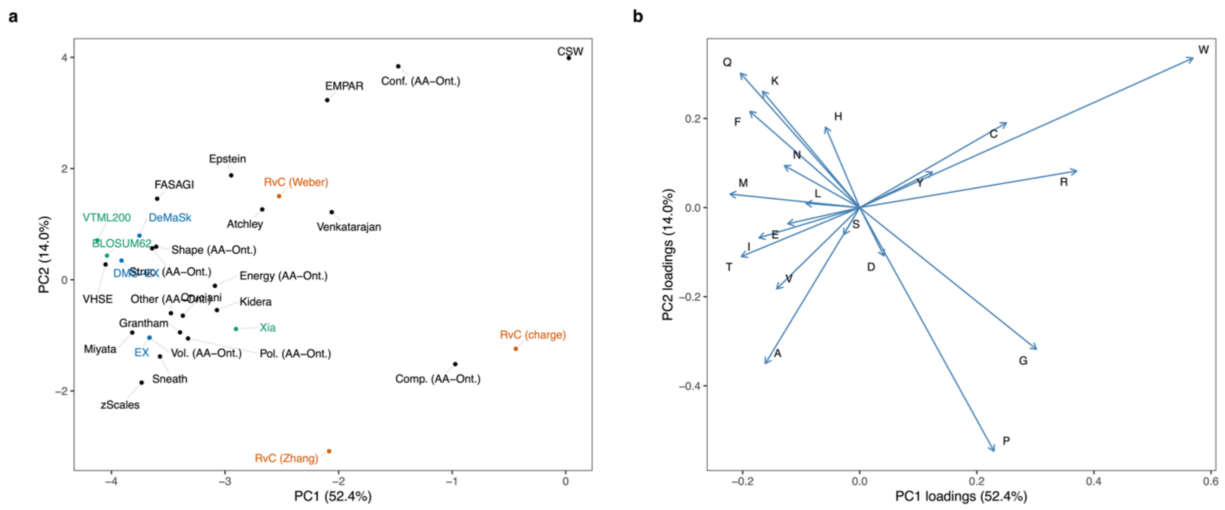

Supplementary Figure 1: Principal Components Analysis of all 30 compared measures, based on Euclidean distances between mean exchangeabilities per amino acid. (a) First two principal components (PCs), with measures indicated. See Methods section for all acronyms. (b) Same analysis as in panel a but displaying the loadings that each amino acid contributes to the separation. Percentage of variance explained by each PC is indicated in parentheses. Measure grouping colours match those in Figure 1.

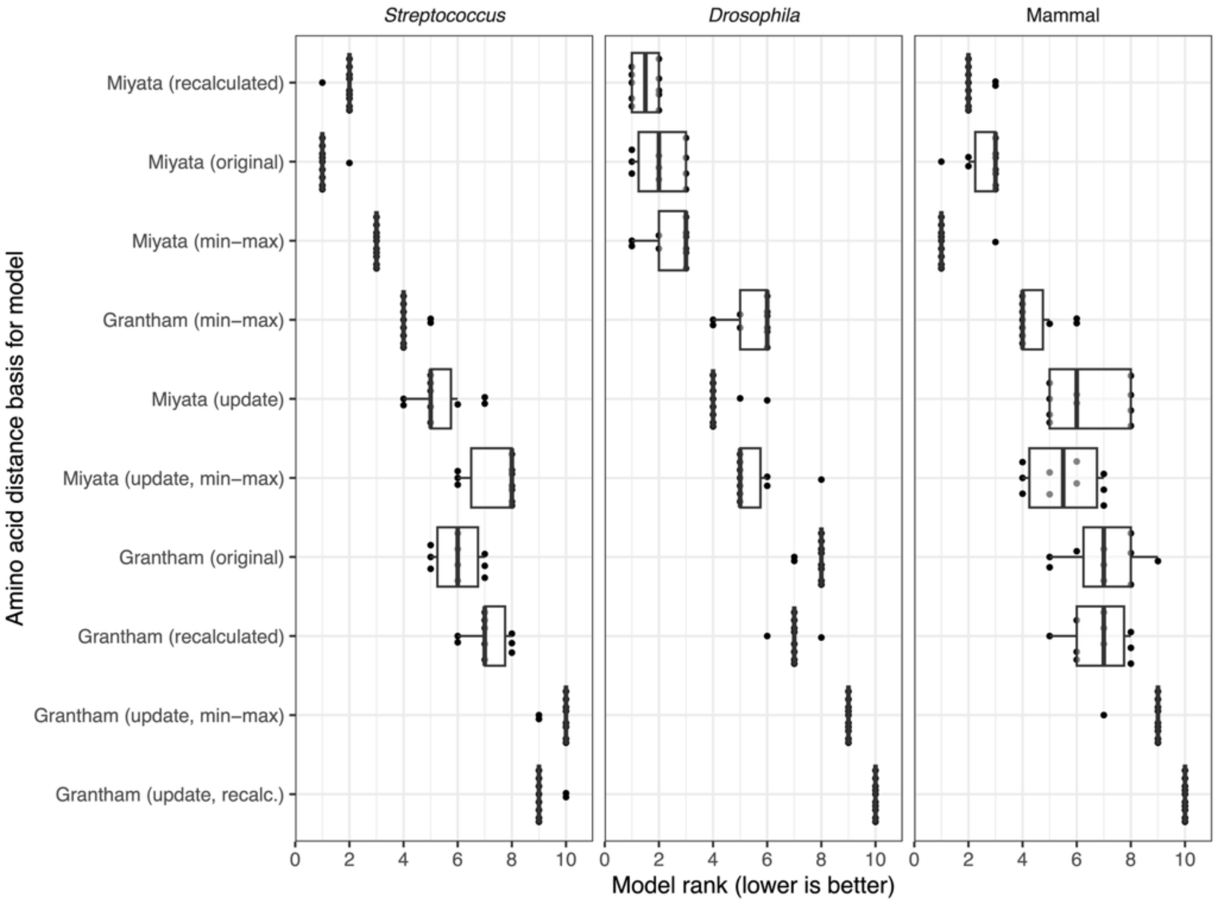

Supplementary Figure 2: Model rank results, as in main text figures, but for variants of Grantham's and Miyata's amino acid distances.

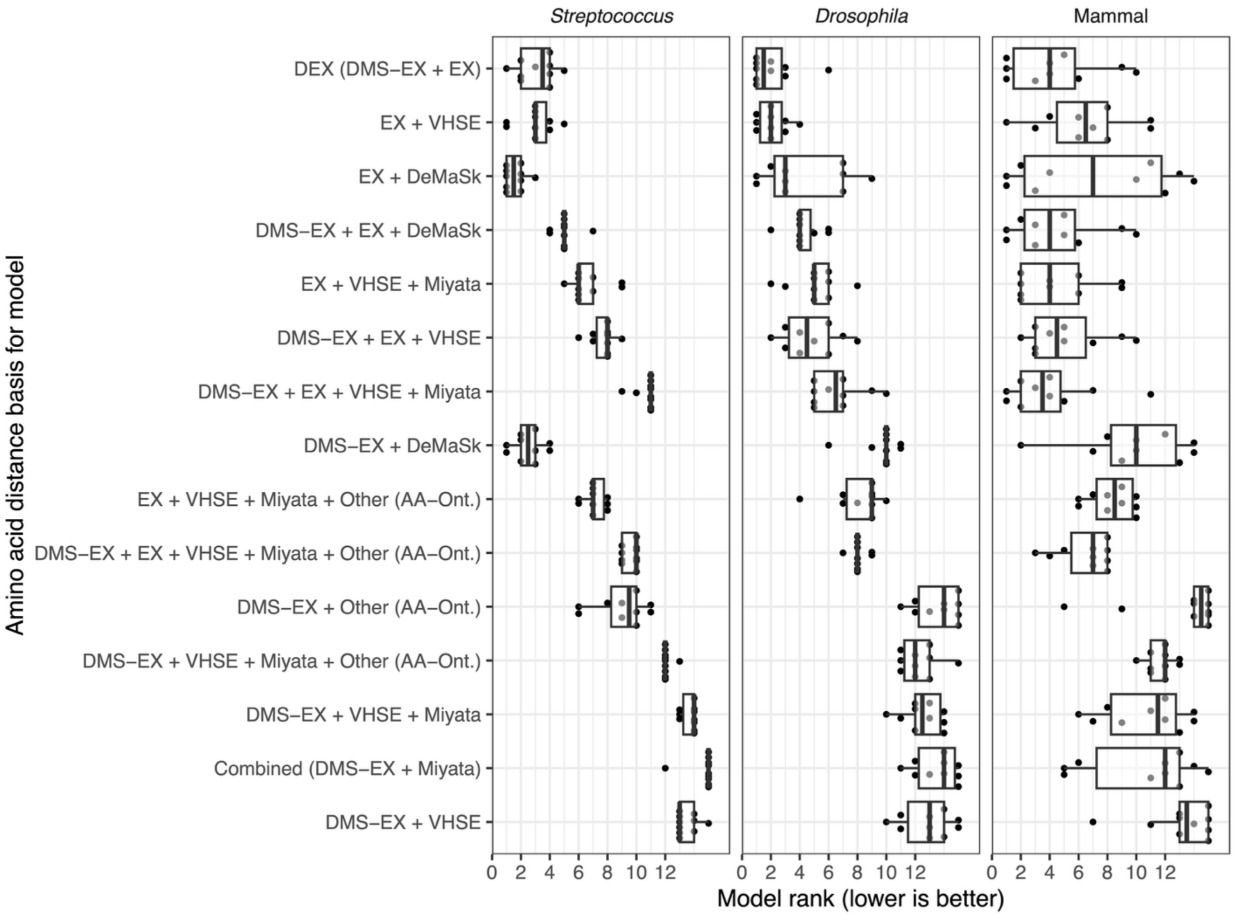

Supplementary Figure 3: Model rank results but for combined amino acid distance measures produced with the DISTATIS approach. This analysis was conducted on an independent set of 10 alignments compared to Figure 2 (i.e., the same set as displayed in Figure 3).

Supplemental Table 1: Deep mutational scanning (DMS) datasets from the ProteinGym database used to define our DMS-EX measure

ProteinGym DMS dataset ID	Source organism	Protein name	Selection assay	Article DOI
**PTEN_HUMAN_Mighell_2018**	*Homo sapiens*	Phosphatidylinositol 3,4,5-trisphosphate 3-phosphatase and dual-specificity protein phosphatase PTEN	Growth	10.1016/j.ajhg.2018.03.018
**MSH2_HUMAN_Jia_2020**	*Homo sapiens*	DNA mismatch repair protein Msh2	Drug resistance	10.1016/j.ajhg.2020.12.003
**MLAC_ECOLI_MacRae_2023**	*Escherichia coli*	Intermembrane phospholipid transport system binding protein MlaC	Cell growth	10.1016/j.jbc.2023.104744
**PHOT_CHLRE_Chen_2023**	*Chlamydomonas reinhardtii*	Phototropin	Fluorescence	10.1021/acssynbio.2c00662
**LGK_LIPST_Klesmith_2015**	*Lipomyces starkeyi*	Levoglucosan kinase	Growth	10.1021/acssynbio.5b00131
**AMIE_PSEAE_Wrenbeck_2017**	*Pseudomonas aeruginosa*	Aliphatic amidase	Enzyme function	10.1038/ncomms15695
**PPARG_HUMAN_Majithia_2016**	*Homo sapiens*	Peroxisome proliferator-activated receptor gamma	Expression of CD36	10.1038/ng.3700
**CBPA2_HUMAN_Tsuboyama_2023_1O6X**	*Homo sapiens*	Carboxypeptidase A2	Stability	10.1038/s41586-023-06328-6
**C6KNH7_9INFA_Lee_2018**	Influenza A virus (A/Perth/16/2009 (H3N2))	Hemagglutinin	Viral replication	10.1073/pnas.1806133115
**RNC_ECOLI_Weeks_2023**	*Escherichia coli*	Ribonuclease 3	Fluorescence	10.1093/molbev/msad047
**KKA2_KLEPN_Melnikov_2014**	*Klebsiella pneumoniae*	Aminoglycoside 3'-phosphotransferase	Growth	10.1093/nar/gku511
**OXDA_RHOTO_Vanella_2023_activity**	*Rhodotorula gracilis*	D-amino-acid oxidase	Fluorescence	10.1101/2023.02.24.529916
**POLG_DEN26_Suphatrakul_2023**	Dengue virus type 2 (strain Thailand/16681/1984) (DENV-2)	Flavivirus NS5	Viral replication	10.1101/2023.03.07.531617
**PRKN_HUMAN_Clausen_2023**	*Homo sapiens*	E3 ubiquitin-protein ligase parkin	Protein stability	10.1101/2023.06.08.544160
**MET_HUMAN_Estevam_2023**	*Homo sapiens*	Hepatocyte growth factor receptor	Cell growth	10.1101/2023.08.03.551866
**UBC9_HUMAN_Weile_2017**	*Homo sapiens*	SUMO-conjugating enzyme UBC9	Yeast growth	10.15252/msb.20177908
**A4D664_9INFA_Soh_2019**	Influenza A virus (A/green-winged teal/Ohio/175/1986 (H2N1))	Polymerase basic protein 2	Viral replication	10.7554/eLife.45079
**A4GRB6_PSEAI_Chen_2020**	*Pseudomonas aeruginosa*	Beta-lactamase VIM-2	Drug resistance	10.7554/eLife.56707

This information was lightly modified from the ProteinGym database metadata provided for all datasets. Note that the DMS dataset ID includes the first author's name and year the data was available, which in several cases is the preprint release year rather than the publication year.

Supplemental Table 2: Amino acid dissimilarity/similarity measures compared in this study

Measure	Category	Brief description	Reference (in main text)
**EX**	Experimental	Exchangeability based on functional impacts of induced substitutions across 12 proteins.	Yampolsky & Stoltzfus 2005
**DMS-EX**	Experimental	New measure of experimental exchangeability computed from deep mutational scanning (DMS) datasets. Not to be confused with the best-performing measure we produced, DEX.	This study
**DEX**	Experimental	DISTATIS-based consensus of EX and DMS-EX; top-performing measure overall in our study.	This study
**DeMaSk**	Experimental	Exchangeability based on rank-transformed substitution impacts across DMS datasets.	Munro & Singh 2021
**BLOSUM62**	Substitution-based	Log-odds substitution matrix based on observed substitutions between sequences with ≤62% identity. Confounded with mutational biases.	Henikoff & Henikoff 1992
**VTML200**	Substitution-based	Substitution matrix derived from a maximum-likelihood model of evolutionary divergence.	Müller et al. 2002
**Xia**	Substitution-based	Distance based on observed amino acid neighbour frequencies in protein sequences.	Xia & Xie 2002
**RvC (Zhang)**	Radical vs. conservative	Binary classification of substitutions as radical or conservative based on polarity and volume differences.	Zhang 2000
**RvC (Weber)**	Radical vs. conservative	Binary classification of substitutions as radical or conservative based on polarity and volume differences, but with distinct groupings from the above.	Weber & Whelan 2019
**RvC (charge)**	Radical vs. conservative	Binary classification based on charge differences between amino acids.	Zhang 2000
**Grantham**	Physicochemical/structural	Distance based on composition, polarity, and molecular volume.	Grantham 1974
**Miyata**	Physicochemical/structural	Distance based on polarity and molecular volume.	Miyata et al. 1979
**Sneath**	Physicochemical/structural	Proportion of 134 physicochemical characteristics not shared between two amino acids.	Sneath 1966
**Epstein**	Physicochemical/structural	Coefficient of difference based on polarity and size.	Epstein 1967
**EMPAR**	Physicochemical/structural	Dissimilarity based on topological and physicochemical features.	Rao 1987
**CSW**	Physicochemical/structural	Similarity based on pairwise comparisons of backbone dihedral angle distributions across 102 crystal structures.	Kolaskar & Kulkarni-Kale 1992
**Cruciani**	Physicochemical/structural	Distance derived from three scaled principal component scores.	Cruciani et al. 2004
**FASAGI**	Physicochemical/structural	Distance based on six components representing diverse amino acid characteristics.	Liang & Li 2008

Measure	Category	Brief description	Reference (in main text)
**Kidera**	Physicochemical/structural	Distance derived from ten orthogonal factors based on multivariate analysis of physicochemical properties.	Kidera et al. 1985
**zScales**	Physicochemical/structural	Distance from five factors based on physicochemical observations including chromatography and nuclear magnetic resonance data.	Sandberg et al. 1998
**VHSE**	Physicochemical/structural	Distance from eight principal components of hydrophobic, steric, and electronic properties.	Mei et al. 2005
**Atchley**	Physicochemical/structural	Distance from five scaled factors summarizing 54 amino acid features.	Atchley et al. 2005
**Venkatarajan**	Physicochemical/structural	Distance derived from five factors representing 237 physicochemical properties.	Venkatarajan & Braun 2001
**Vol. (AA-Ont.)**	Physicochemical/structural	Distance derived from principal coordinates of volume-related scales in AAontology.	Breimann et al. 2024
**Pol. (AA-Ont.)**	Physicochemical/structural	Distance derived from principal coordinates of polarity-related scales in AAontology.	Breimann et al. 2024
**Struc. (AA-Ont.)**	Physicochemical/structural	Distance derived from principal coordinates of structure/activity-related scales in AAontology.	Breimann et al. 2024
**Energy (AA-Ont.)**	Physicochemical/structural	Distance derived from principal coordinates of energy-related scales in AAontology.	Breimann et al. 2024
**Conf. (AA-Ont.)**	Physicochemical/structural	Distance derived from principal coordinates of conformation-related scales in AAontology.	Breimann et al. 2024
**Shape (AA-Ont.)**	Physicochemical/structural	Distance derived from principal coordinates of shape-related scales in AAontology.	Breimann et al. 2024
**Comp. (AA-Ont.)**	Physicochemical/structural	Distance derived from principal coordinates of composition-related scales in AAontology.	Breimann et al. 2024
**Other (AA-Ont.)**	Physicochemical/structural	Distance derived from principal coordinates of miscellaneous scales in AAontology.	Breimann et al. 2024
